# Study on the Risk of Phosphorus Leaching in Dryland from Typical Purple-Soil Regions and Its Control Mechanisms

**DOI:** 10.3390/biology15181658

**Published:** 2026-09-19

**Authors:** Xiaosong Yang, Jingwen Yu, Yanfen Wang, Yiming Zhao, Kun Wang, Xiaofeng Lu, Lan Zhang

**Affiliations:** 1 State Key Laboratory of Environmental Criteria and Risk Assessment, National Engineering Laboratory of Lake Water Pollution Control and Ecological Restoration Technology, Chinese Research Academy of Environmental Sciences, Beijing 100012, China; yangxiaosong15@mails.ucas.ac.cn (X.Y.); 19863962513@163.com (Y.W.); 15776814368@163.com (Y.Z.); wangkun@craes.org.cn (K.W.); 2 Yangtze Ecology and Environment Co., Ltd., Wuhan 430014, China; wei_jingwen@ctg.com.cn; 3 College of Water Conservancy, Shenyang Agricultural University, Shenyang 110866, China

**Keywords:** phosphorus, biochar, silicon-based conditioner, metagenomics, functional genes

## Abstract

Phosphorus (P) leaching from dryland from purple soils poses a significant risk to water quality, yet effective mitigation strategies and their underlying microbial mechanisms remain poorly understood. This study aimed to evaluate the efficacy of biochar (B), a silicon-based conditioner (Si), and their combination (BSi) in controlling P leaching, hypothesizing that B would immobilize P while Si would mobilize it. The indoor soil column leaching experiments were conducted with four treatments (CK, B, Si, BSi), measuring leachate P fractions and soil P forms, and employed metagenomic sequencing combined with partial least-squares path modeling (PLS-PM) and Bayesian structural equation modeling (BSEM) to explore microbial functional mechanisms. Results showed that B alone reduced cumulative leaching of inorganic P (IP), organic P (OP), and total P (TP) by a range of 5.4–6.3%, while increasing available phosphorus (Olsen-P) by 39.4% in the surface layer. Si and BSi promoted leaching, with BSi reducing available P sharply, despite raising TP. Metagenomic analysis revealed that B suppressed subsurface IP solubilization genes (e.g., *gcd*, *ppx*) and optimized OP mineralization, whereas Si inhibited mineralization via reducing key microbial taxa. BSEM further identified water-soluble P (Water-P) and total nitrogen (TN) as direct positive drivers of inorganic P dissolution. Collectively, the key biological mechanisms for leaching reduction involve inhibiting subsurface IP solubilization, optimizing surface OP mineralization, and enhancing P transport/starvation responses. Collectively, biochar applied alone offers the optimal balance between P retention and crop-available P supply in dryland purple soils, and provides mechanistic insights—through functional gene profiling—that can inform the design of more sustainable P fertilization and leaching control practices.

## Simple Summary

Phosphorus leaching from dryland purple soils threatens water quality. This study tested biochar, a silicon-based conditioner, and their combination to reduce this risk. Biochar alone performed best—cutting phosphorus leaching by 5–6% and increasing plant-available phosphorus by 40% in surface soil. Silicon and the combined treatment worsened leaching. Genetic analysis showed biochar suppresses phosphorus-dissolving microbial genes in deeper layers while enhancing plant-accessible phosphorus path-ways. Biochar offers a practical solution for balancing agricultural productivity and environmental protection.

## 1. Introduction

Phosphorus (P) leaching from dryland purple soils poses a significant risk to water quality, yet effective mitigation strategies and their underlying mechanisms remain poorly understood. Purple soils are developed from P-rich parent materials through rapid weathering, and are characterized by a sandy texture, loose structure, and strong infiltration capacity [1,2,3,4]. Under dryland cultivation, long-term surface application of phosphate fertilizers leads to P enrichment in the topsoil, while frequent wetting–drying cycles and intensive rainfall accelerate the downward migration of soluble phosphorus to deeper soil layers, posing a potential risk of groundwater and adjacent surface water contamination [5,6,7,8].

The P behavior in dryland soils differs markedly from that in paddy fields. First, drylands are predominantly aerobic, where iron and aluminum oxides exhibit strong phosphorus fixation, yet frequent wetting–drying cycles promote the release of occluded P and the generation of water-soluble P [9,10,11]. Second, dryland fertilization is mainly based on surface application of basal fertilizers, leading to P accumulation in the surface layer; meanwhile, tillage disturbance and rainfall infiltration further accelerate the downward migration of surface P. Third, compared with forest lands, drylands lack deep-root biological retention and litter cover protection, resulting in more direct phosphorus export pathways [12,13,14]. Purple soils in the hilly regions of southwestern China are important dry-farming soil types, and the complex climatic conditions make P susceptible not only to surface runoff losses but also to vertical leaching through soil profiles, constituting a persistent source of risk for regional water eutrophication [15]. Current research on mitigation measures for P leaching in purple-soil drylands remains insufficient, particularly lacking comparative evaluations and mechanistic insights into different conditioners within the soil–water–microorganism coupling system.

Biochar (B) and silicon-based conditioner (Si) are soil P-regulating materials that have attracted widespread attention in recent years [16,17,18]. B, with its abundant porous structure, high specific surface area, and surface oxygen-containing functional groups, can effectively adsorb and immobilize soluble P and promote its transformation into more stable forms [19,20,21]. Si can influence the dissolution–migration balance of P by competing with iron and aluminum oxides for adsorption sites and by raising soil pH [22]. However, the interactive effects of these two conditioners in dryland soils remain unclear. In particular, whether the activation effect of silicon actually exacerbates leaching under the strong infiltration conditions of drylands and whether B can offset such negative impacts, lack empirical evidence [23]. Existing studies on biochar and silicon co-application report contradictory outcomes regarding P mobility. Silicon can mobilize P by competing with Fe/Al oxides for sorption sites, whereas biochar can immobilize P through its porous structure and surface functional groups. However, the net effect depends on boundary conditions such as soil pH, Fe oxide mineralogy, and hydrological regime [19,22]. These contrasting findings highlight that synergistic or antagonistic interactions are context-specific. Nevertheless, no study has systematically compared these amendments in dryland purple soils, nor investigated the microbial functional gene responses. More importantly, P migration and transformation are essentially regulated by microbial-driven gene networks involved in inorganic P (IP) solubilization, organic P (OP) mineralization, P transport and uptake, and P starvation regulation [24,25]. Yet, most existing studies are confined to chemical fraction analysis, and very few have employed metagenomic approaches to reveal how conditioners affect P leaching in dryland by altering functional gene expression.

We hypothesized that biochar would effectively reduce P leaching by immobilizing soluble P through its porous structure and functional groups, whereas silicon would mobilize P via competitive desorption with Fe/Al oxides, and that the combined application would result in an antagonistic rather than synergistic outcome due to the dominance of Si mobilization under dryland aerobic conditions. Therefore, this study takes typical dryland from purple-soil region as the research object, conducts indoor soil column leaching experiments, and sets up four treatments: control (CK), biochar (B), silicon-based conditioner (Si), and their combined application (BSi). The effects of different conditioners on P-leaching amounts and profile fraction distributions were compared systematically, and the response patterns of key phosphorus-cycling functional genes (e.g., *gcd*, *ppx*, *pstA/B*, *ugpB/C*, *phoB/R*, etc.) were analyzed in combination with metagenomic sequencing. Partial least-squares path modeling (PLS-PM) and Bayesian structural equation modeling (BSEM) were utilized to elucidate the regulatory network among environmental factors (Olsen-P, Water-P, TN), functional genes, and P-cycling processes. The results are expected to provide targeted conditioning strategies for P-leaching risk control in drylands from purple-soil regions, and to offer a scientific basis for achieving the synergistic management of P retention and fertilizer conservation and P-supply efficiency enhancement.

## 2. Methods and Materials

### 2.1. Study Area and Sampling Sites

The study area is located in the Quanmin Reservoir Watershed (106.49° E–106.57° E, 30.55° N–30.61° N), Sichuan Province, within the purple hilly region of the eastern Sichuan Basin, covering 425.5 km^2^. The watershed extensively exposes Mesozoic Jurassic to Cretaceous purple sandstones and mudstones. Driven by a mid-subtropical humid monsoon climate (mean annual temperature range: 17–18 °C, precipitation range: 1000–1200 mm, with >50% of annual rainfall concentrated in summer storms), rapid physical weathering has developed calcareous purple soils. P-leaching losses triggered by heavy rainfall have become an important risk for regional water eutrophication, particularly during the summer rainstorm period. For the specific soils used in this study, the surface layer (0–20 cm) had a clay content of approximately 210 g kg^−1^ and a loam to clay loam texture, while the subsurface layer (20–40 cm) contained approximately 190 g kg^−1^ clay (Table S1). These textural characteristics—moderate to relatively high clay content combined with a sandy loam to loam matrix—contribute to the strong infiltration capacity and the associated P-leaching risk observed in this dryland purple-soil system.

**Figure 1:**
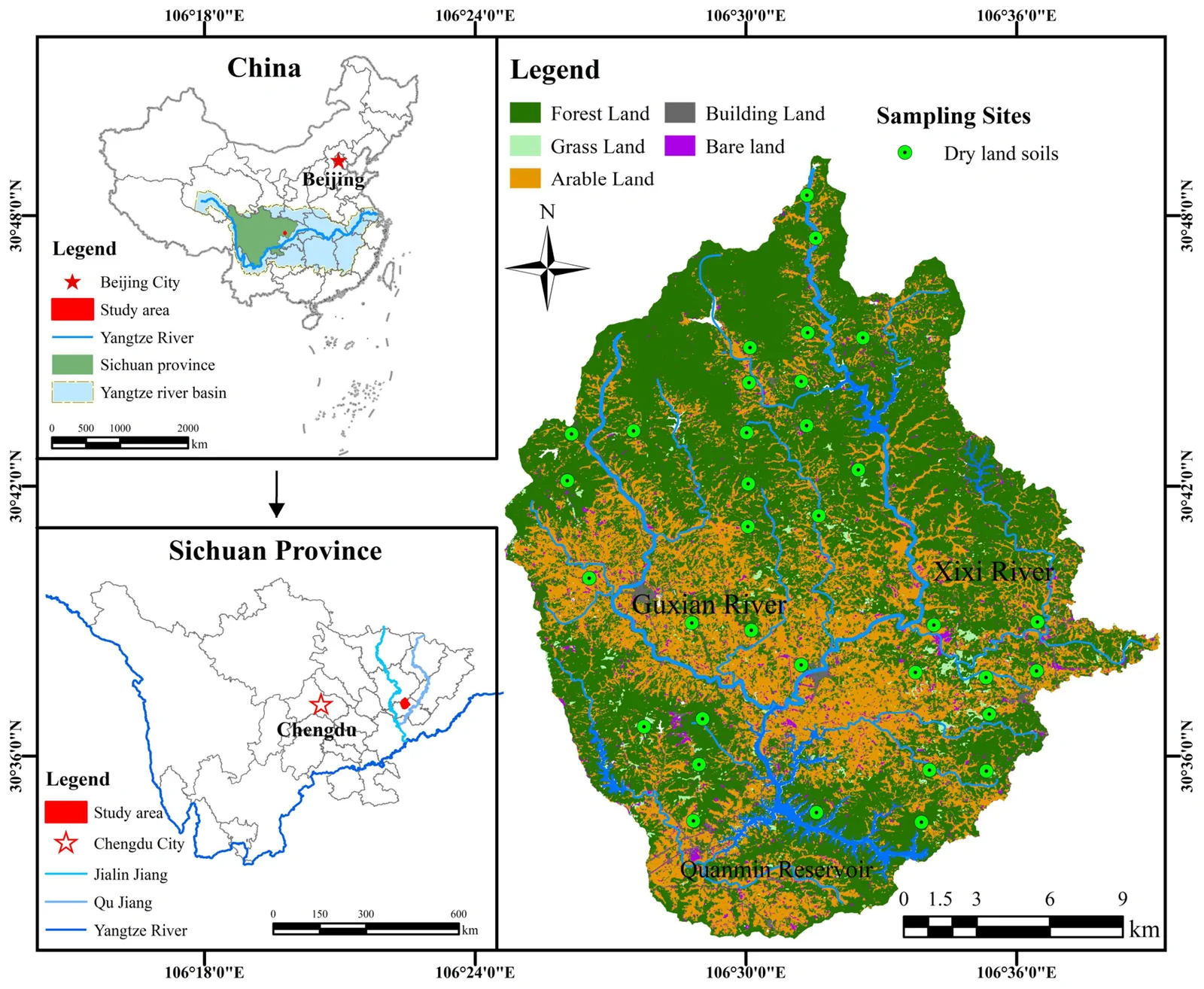
Sampling sites in the Quanmin Reservoir Watershed.

A soil sampling survey was conducted across dryland within the study area in October 2025. A total of 68 soil samples were collected from 34 sampling sites in dryland at two depths in the ranges 0–20 cm (surface) and 20–40 cm (subsurface) (Figure 1). Field sampling used GPS for coordinate positioning. At each sampling point, three sub-sampling positions were arranged in a 1 m × 1 m × 1 m equilateral triangle, from which undisturbed soil samples of the corresponding depths were collected. The three subsamples at the same depth were thoroughly mixed, sealed in self-sealing bags, and transported to the laboratory. Upon arrival, visible gravel and undecomposed plant residues were removed, and the samples were air-dried in a ventilated, shaded area. After air-drying, roots and impurities were removed, and the soils were successively passed through 2 mm, 1 mm, and 0.15 mm nylon sieves.

Soil for the leaching experiment was collected from Hongmiao Temple Village (106°26′22.377″ E, 30°41′0.97″ N), within the Quanmin Reservoir Watershed. This area is under long-term maize cultivation, and the soil type is purple soil. Soil sampling was carried out separately for the 0–20 cm and 20–40 cm range layers. The collected soil samples were air-dried in layers in a ventilated indoor area, ground, and passed through a 2 mm sieve. A portion of soil from each layer was taken for the determination of basic physicochemical properties, and the remaining soil was used for the soil column packing experiment. The physicochemical properties of the tested soils are presented in Table S1.

### 2.2. Soil Column Leaching Experiments

Biochar was produced from bamboo feedstock pyrolyzed at 500 °C for 8 h. The biochar had a total nitrogen (TN) content of 15.68 g kg^−1^, dissolved organic carbon (DOC) content of 1250.00 mg kg^−1^, total phosphorus (TP) content of 0.83 g kg^−1^, available phosphorus (Olsen-P) content of 61.24 mg kg^−1^, and water-soluble P (Water-P) content of 38.78 mg kg^−1^ (Table S2). The Olsen-P and Water-P values for biochar (Table S2) were determined using soil extractants (0.5 M NaHCO_3_ and water). Although these methods are widely used in biochar studies for estimating extractable P, they may not fully recover all P pools (e.g., Ca/Mg-bound P) because biochar is more akin to organic amendments than to soil. Therefore, these values should be considered as comparative operational indices rather than absolute measures of total bioavailable P in biochar. The silicon-based conditioner used in this study was passed through a 2 mm sieve and thoroughly mixed. The conditioner had a TN content of 2.10 g kg^−1^, DOC content of 0.80 mg kg^−1^, TP content of 0.51 g kg^−1^, Olsen-P content of 45.44 mg kg^−1^, and Water-P content of 51.49 mg kg^−1^.

Four treatments were established: (1) CK: blank control (no amendment); (2) B: biochar applied at 30 t ha^−1^ mixed with soil; (3) Si: silicon-based conditioner applied at 30 t ha^−1^ mixed with soil; and (4) BSi: combined application of biochar and silicon-based conditioner at 30 t ha^−1^ each mixed with soil. Each treatment received 178.5 kg N ha^−1^ and 90 kg P_2_O_5_ ha^−1^, with three replicates per treatment, as shown in Table S3.

A layered soil column leaching system was employed, using transparent organic glass columns with a length of 30 cm and an inner diameter of 6.0 cm as the leaching apparatus (Figure S1) to simulate water and nutrient transport in natural soil. Based on the bulk density of each soil layer, the required soil packing masses were calculated: the surface soil (0–20 cm) had a bulk density of 1.22 g cm^−3^, requiring 689.89 g; the subsurface (20–40 cm) layer had a bulk density of 1.24 g cm^−3^, requiring 701.2 g. The packing procedure was as follows: first, the 20–40 cm soil layer was packed. A 200-mesh nylon sieve was placed at the bottom of the column to prevent soil particle loss during leaching. Then, 20 g of coarse quartz sand was added to enhance bottom permeability, followed by 701.2 g of subsurface soil. Subsequently, an additional section of organic glass column was attached, with a sealing gasket and petroleum jelly applied at the joint to ensure a leak-tight seal. Next, according to the experimental design, the biochar or silicon-based conditioner was thoroughly mixed with the surface soil; half of the mixture was added to the column, and the remaining half was mixed with 0.212 g of calcium superphosphate before being added. Finally, a 2 mm layer of coarse quartz sand was placed on the top of the soil surface to prevent disturbance from the simulated rainfall.

One week before the experiment, ultrapure water was added to saturate the soil. The leaching experiment was conducted using artificial simulated rainfall. Each rainfall event consisted of 100 mL of water, equivalent to an infiltration of 35 mm, which corresponds to a heavy rain intensity according to meteorological standards. The experiment began in December 2025, with the first leachate collection. The leaching events were performed at 12-day intervals, for a total of six simulated rainfall events. The volume of each leachate was recorded, and the concentrations of TP, OP, and IP in the leachate were determined. After the experiment, soil samples were collected from both the surface and subsurface layers to determine pH, TN, TP, Olsen-P, and Water-P contents, and metagenomic analysis was also conducted.

### 2.3. Physicochemical Analysis

Olsen-P in soil was determined by extraction with 0.5 mol L^−1^ NaHCO_3_ (pH 8.5). Specifically, 2 g of air-dried soil (<2 mm) was placed in a 50 mL centrifuge tube, and 0.5 mol L^−1^ NaHCO_3_ solution (pH 8.5) was added at a soil-to-solution ratio of 1:20. The mixture was shaken for 30 min at 25 °C, then filtered through phosphorus-free filter paper. The P concentration in the filtrate was measured by the molybdenum blue colorimetric method. Water-P in soil was determined using ultrapure water as the extractant at a soil-to-water ratio of 1:5. The mixture was shaken for 30 min at 25 °C and filtered through P-free filter paper, and the P in the filtrate was also measured by the molybdenum blue colorimetric method. TN in soil was determined using a TC/TN analyzer (Multi N/C3000, Analytik Jena, Jena, Germany). TP in the leachate was determined by the potassium persulfate digestion-molybdenum blue colorimetric method. The procedure was as follows: 20 mL of leachate was mixed with 4 mL of 50 g L^−1^ potassium persulfate solution and digested at 121 °C for 30 min. After digestion, the volume was made up to 25 mL with ultrapure water, and the P concentration was determined by the molybdenum blue colorimetric method. IP in the leachate was directly measured by the molybdenum blue colorimetric method. OP in the leachate was calculated as the difference between TP and IP.

### 2.4. Metagenomic Sequencing

Metagenomic sequencing was performed using paired-end sequencing on the Illumina NovaSeq platform (Wekemo Tech Group Co., Ltd. Shenzhen, China). The raw data were filtered with Trimmomatic to remove sequences with quality scores below 20, sequences containing ≥10% ambiguous bases (N), or sequences shorter than 75 bp, yielding high-quality reads. Subsequently, the clean reads were assembled using Megahit (v1.2.9), and contigs longer than 300 bp were retained. Open reading frames (ORFs) in these contigs were predicted using METAProdigal (v2.6.3), and then clustered with CD-HIT (at 95% identity and 90% coverage) to construct a non-redundant gene set. This gene set was aligned to the phosphorus functional gene database (PCycDB) (https://github.com/ZengJiaxiong/Phosphorus-cycling-database, accessed on April 2026) for functional annotation. Based on the classification of soil phosphorus-cycling microbial functional genes in the KEGG database (https://www.genome.jp/kegg/, accessed on April 2026) and previous studies, the functional genes in this study were broadly categorized into four groups: IP solubilization genes, OP mineralization genes, P-uptake and transport genes, and starvation regulation regulatory genes. Meanwhile, the gene set was also aligned to the NR database for taxonomic annotation.

### 2.5. Data Analysis

Partial least-squares path modeling (PLS-PM) was constructed using R 4.5.2 (R Studio) to integrate soil properties, P-cycling microbial abundance at the genus level, and P-cycling functional genes under different treatments, establishing a path model of “environmental factors–P-cycling microorganisms–P-cycling functional gene groups” to analyze the direct or indirect effects of environmental factors on each phosphorus-cycling process. A model goodness of fit (GOF) ≥ 0.36 was considered excellent, and the following criteria were simultaneously required: core endogenous variables with R^2^ ≥ 0.33, key paths with *p* < 0.05, and Q^2^ > 0. On this basis, a Bayesian structural equation model (BSEM) was constructed to specifically analyze the relationships between soil environmental factors (Olsen-P, Water-P, and TN) and the functional gene groups corresponding to the four P-cycling processes described above. It should be noted that PLS-PM was fitted separately for each treatment with limited observations (n = 6 per treatment, combining replicates and soil layers). Therefore, the treatment-specific path coefficients were interpreted as exploratory and were not used for definitive statistical inference. The primary inferential conclusions were drawn from the BSEM, which included all treatments simultaneously (n = 24), and its robustness was verified by convergence diagnostics.

## 3. Results and Discussion

### 3.1. Analysis of Distribution Characteristics of P in Study Areas

**Figure 2:**
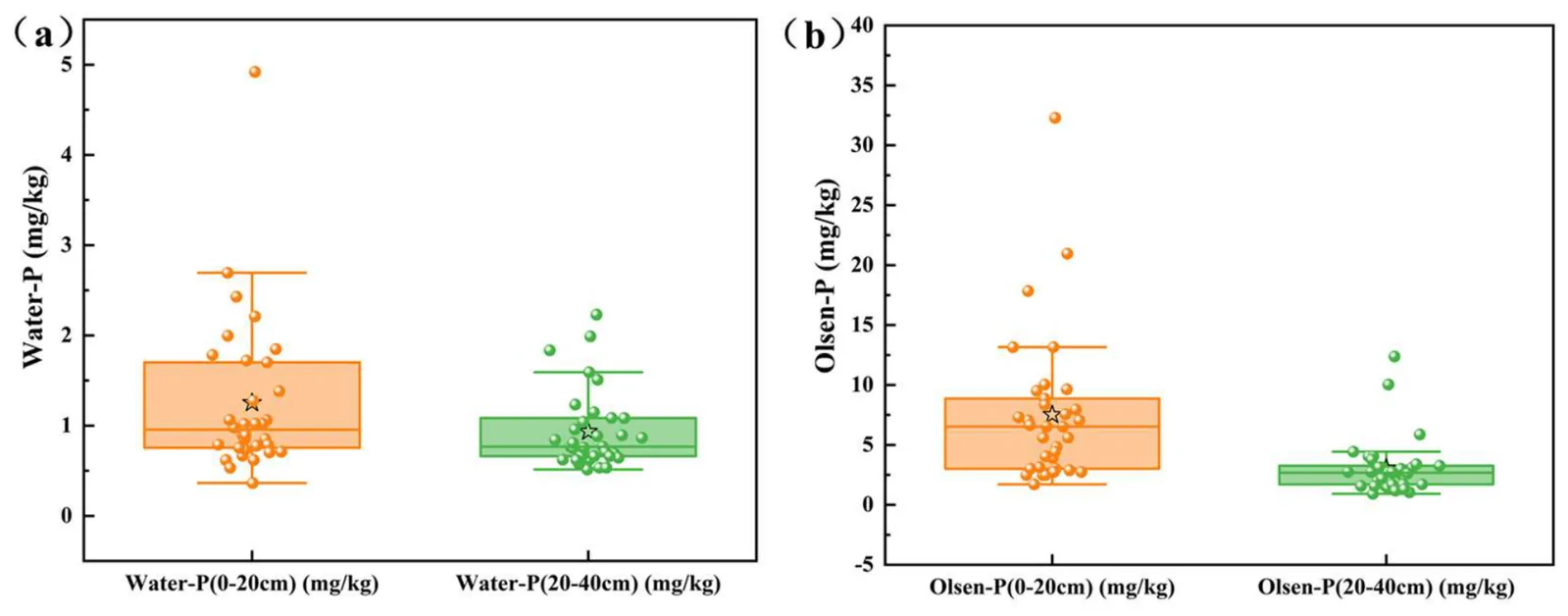
Soil Olsen-P (**a**) and Water-P (**b**) contents in different profiles. The black pentagrams in the figure represent the mean values.

In the study area, Olsen-P was relatively deficient (6.92 mg kg^−1^ in the surface layer and 4.13 mg kg^−1^ in the subsurface layer), and Water-P contents were also low (1.10 mg kg^−1^ and 0.90 mg kg^−1^, respectively), as shown in Figure 2. Previous studies have reported that the average Olsen-P content in surface purple soils from some regions was 15.34 mg kg^−1^, and the subsurface average was 7.2 mg kg^−1^, both higher than the values observed in this study area, confirming the deficiency of Olsen-P in this region. This difference can be attributed to two main factors. First, the P-fertilizer application rate in the study area’s farmland was relatively low, with an intensity of 86.99 kg P_2_O_5_ ha^−1^, significantly lower than the average level of 138.64 kg P_2_O_5_ ha^−1^ in other surrounding purple-soil regions [26]. Second, the land use type in the study area is dominated by forestland, where soils lack exogenous P inputs. Combined with abundant regional precipitation and undulating topography, the strong leaching and hydraulic erosion accelerate the vertical migration and lateral loss of soil phosphorus, further weakening the availability and accumulation capacity of P in soil [27]. The Water-P contents in the surface and subsurface soils of the study area were 1.10 mg kg^−1^ and 0.90 mg kg^−1^, respectively, both higher than the values (0.5–0.83 mg kg^−1^) the similar regions. This is mainly attributed to the high P content of the parent rock in purple-soil regions, combined with long-term intensive application of chemical and organic fertilizers, which has led to continuous P accumulation in soil. In addition, the loose soil structure facilitates the downward migration of surface P with infiltrating water under heavy rainfall, resulting in relatively high Water-P concentrations in both the surface and subsurface layers [28,29].

### 3.2. Characteristics of P-Leaching Solution and P Dynamics in Soil

**Figure 3:**
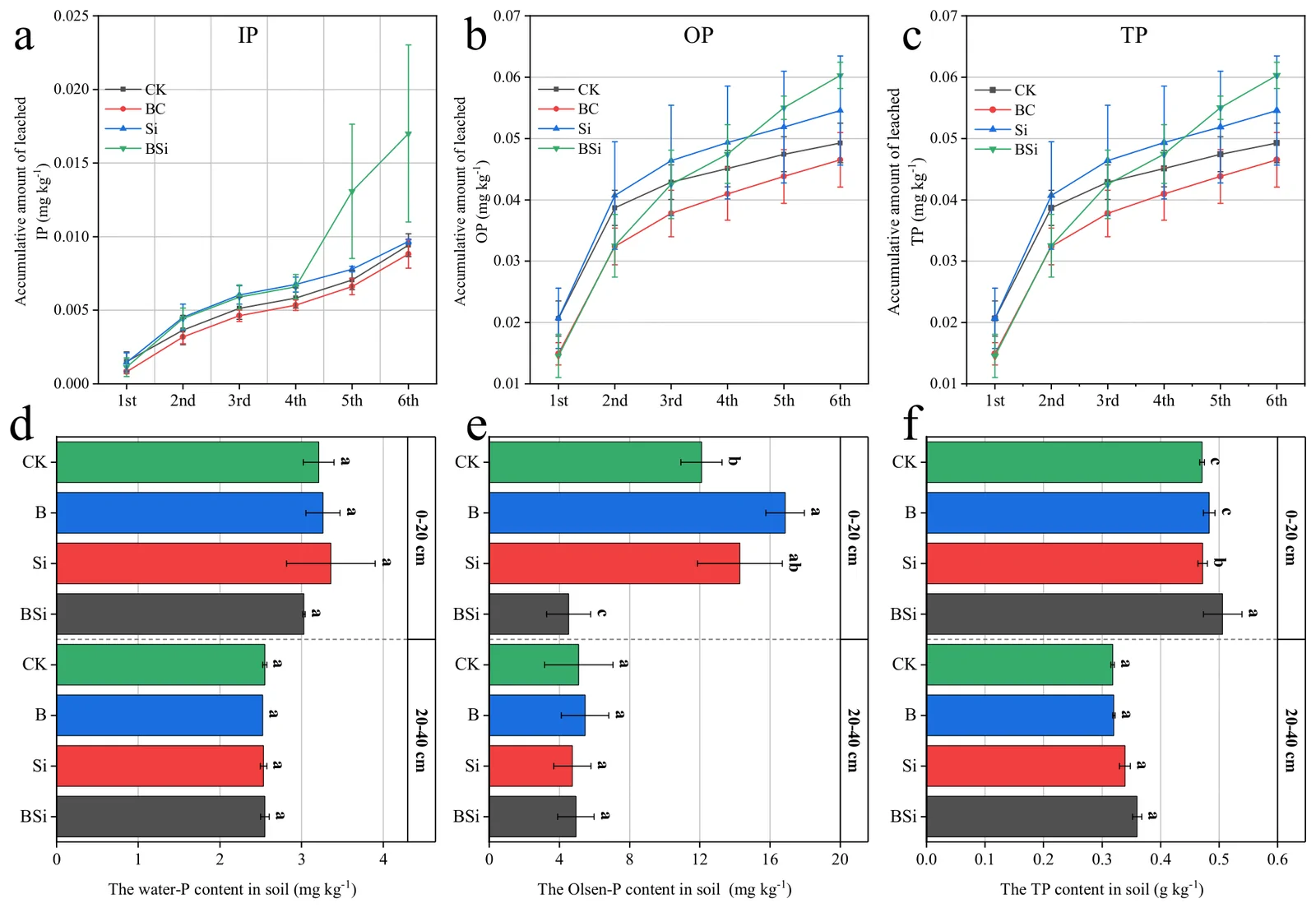
Cumulative leaching losses of IP (**a**), OP (**b**), and TP (**c**) under different treatments over the entire six-event leaching period, and contents of soil Water-P (**d**), Olsen-P (**e**), and TP (**f**) under different treatments. Different lowercase letters indicate significant differences at *p* < 0.05.

After six leaching events, compared with CK, the B treatment significantly reduced the cumulative (i.e., summed over all six events) leaching losses of soil IP, OP, and TP by 6.31%, 5.42%, and 5.59%, respectively (Figure 3a–c). In contrast, Si and BSi treatments promoted leaching, with the BSi treatment showing the highest leaching risk; the cumulative leached amounts of IP, OP, and TP reached 0.017, 0.024, and 0.043 mg/kg, respectively. The overall order of leaching amounts was BSi > Si > CK > B. After leaching, TP and Olsen-P contents showed differentiated responses. For Water-P, the Si treatment had the largest increase in the surface layer (4.59%), while BSi decreased it; in the subsurface layer, both B and Si decreased in Water-P content, with the smallest decrease in B (1.02%). For Olsen-P, the B treatment showed the most prominent increase, raising it by 39.35% in the surface layer and 7.14% in the subsurface layer; however, BSi caused a sharp decrease of 62.58% in surface Olsen-P, and Si also reduced subsurface Olsen-P. For TP, all treatments increased, with BSi showing the largest increase (7.40% in surface and 13.03% in subsurface layers). Overall, the B treatment performed best in both retarding P leaching and maintaining Olsen-P levels; Si was intermediate; BSi, although increasing TP, greatly reduced Olsen-P, posing a risk to crop growth.

P-leaching loss is an important pathway of environmental water pollution. The results showed that the B treatment significantly reduced the leaching amounts of IP, OP, and TP (Figure 3a–c), which is consistent with previous indoor column simulation results [30]. The previous study reported that B significantly reduced the leaching of IP and TP [31], mainly due to the P sorption by the porous structure and high specific surface area of biochar, as well as its ability to induce a transformation of P from more labile to more stable forms. Both the Si and BSi treatments promoted the leaching of IP, OP, and TP (Figure 3a–c). On the one hand, silicates compete with Fe/Al oxides for sorption sites, displacing previously fixed P [22]; on the other hand, Si addition increases soil pH, enhancing the solubility and mobility of phosphate ions [32]. The enhanced leaching under BSi treatment may be attributed to the dominant activation effect of the silicon-based conditioner, and the porous structure of biochar may provide preferential flow paths for P transport, which might increase the risk of P loss. In summary, biochar applied alone exerts a significant and stable inhibitory effect on soil P leaching.

The different treatments significantly affected soil P fractions and their profile distribution (Figure 3d–f), reflecting the distinct mechanisms of biochar and silicon-based conditioners in P transformation and migration. The marked increase in Olsen-P under the B treatment indicates that, while immobilizing part of the Water-P, biochar, with its abundant pore structure and surface oxygen-containing functional groups, effectively increases soil cation exchange capacity, thereby improving soil physicochemical properties and enhancing nutrient adsorption and retention, thus raising soil Olsen-P [33]. The decrease in subsurface Water-P further confirms the retarding effect of biochar on downward P migration, closely related to its adsorption–fixation and physical entrapment [34]. The Si treatment showed a pronounced activation effect on various P fractions in the surface layer but only increased TP in the subsurface layer. This is mainly because, after Si application, surface soil pH increased, while subsurface pH remained lower, limiting silicic acid dissociation and thus leading to more stable P fixation [22]. In the short term, Si promoted P dissolution and redistribution; however, because it could not effectively retard P migration, P tended to move downward with water and accumulate in deeper layers. The BSi treatment increased TP in both layers but decreased surface Olsen-P and Water-P, suggesting a complex interactive effect between biochar and silicon-based conditioner: on one hand, Si activation promoted the migration of part of the labile P to deeper layers or its fixation into more stable forms; on the other hand, biochar sorption and retention redistributed P within the profile, ultimately resulting in lower labile P but higher TP. In summary, biochar application provides the best retardation of soil P leaching without compromising crop growth, but in practical application, a further balance between P retention/fertilizer conservation and P-supply efficiency needs to be carefully considered.

### 3.3. Analysis of Changes in the Relative Abundance of Microbial Functional Genes During P Cycling in Soil

Based on the distinct roles of microbial functional genes in soil P cycling, these genes were classified into four functional groups: IP solubilization genes, OP mineralization genes, P transport and uptake genes, and P starvation regulation genes. After removing functional genes with relative abundances below 0.01‰, a total of 15 phosphorus-cycling-related functional genes were identified. Among them, two genes were associated with IP solubilization, six genes with OP mineralization, five genes with P transport and uptake, and two genes with P starvation regulation. The detailed classification of these phosphorus-cycling functional genes is provided in Table 1.

**Figure 4:**
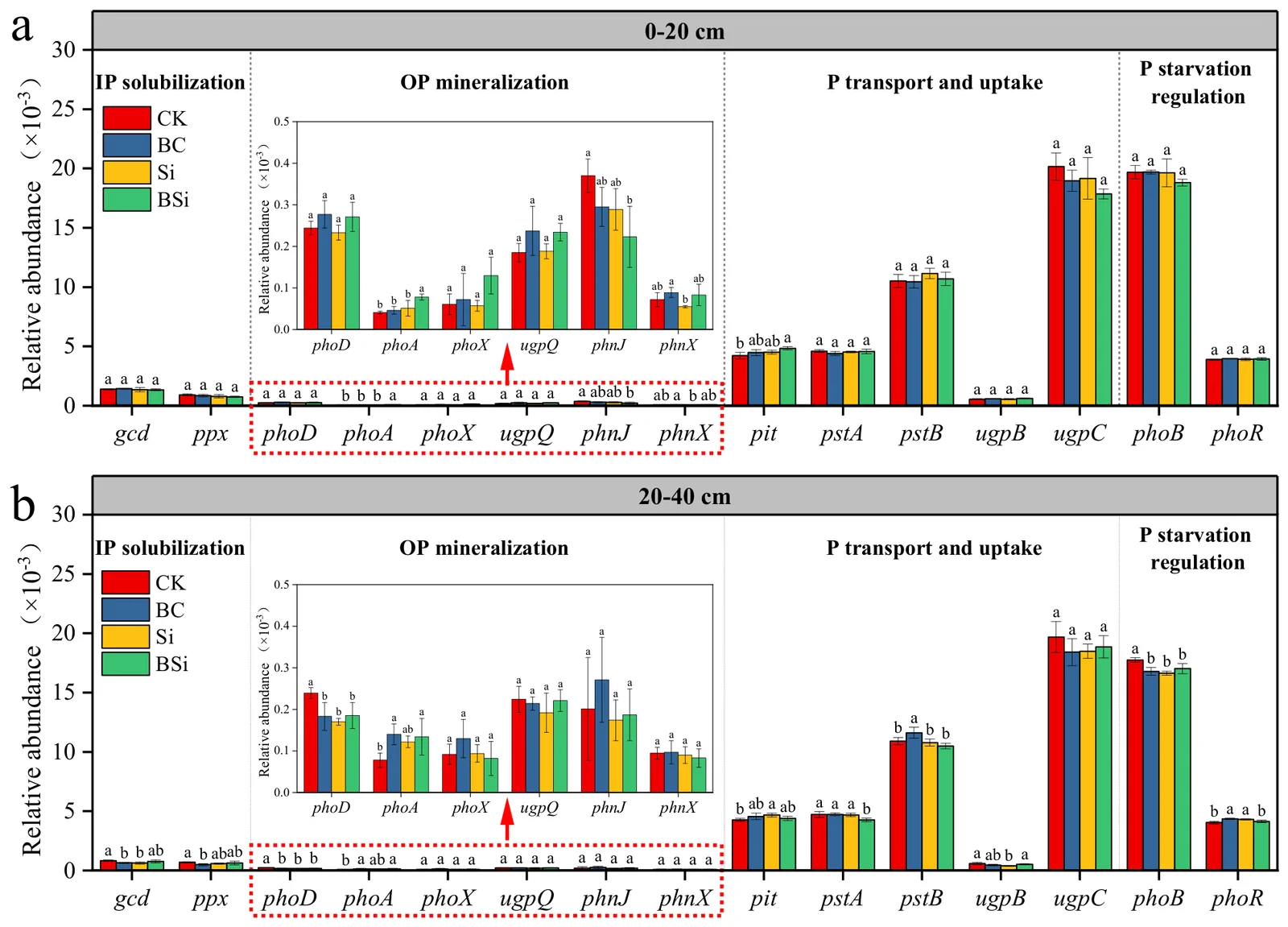
Abundance differences of P-cycling functional genes under different treatments. (**a**). 0–20 cm; (**b**). 20–40 cm. Different lowercase letters indicate significant differences at *p* < 0.05.

Under different treatments, the dominant functional genes in both soil layers were consistently *gcd*, *ppx*, *pit*, *pstA*, *pstB*, *ugpB*, *ugpC*, *phoB*, and *phoR* (relative abundance > 0.5‰) (Figure 4). In the surface layer, compared with CK, the B treatment promoted all stages of the P cycle, favoring an increase in the diversity of P-cycling-related microorganisms. The Si treatment exhibited a general inhibitory effect on the gene abundances associated with each stage of the soil P cycle. The BSi treatment promoted OP mineralization and showed some effectiveness in P fixation, but it reduced plant-available P in the surface layer, which is detrimental to crop growth. In the subsurface layer, compared with CK, all other treatments effectively reduced the abundances of P-cycling-related genes. Regarding IP solubilization, the B treatment performed best, with the lowest abundances of IP solubilization-related genes, indicating a certain inhibitory effect on the IP dissolution process in the subsurface layer, thereby reducing the production of soluble P and achieving the best retardation of P leaching. In terms of OP mineralization, the Si treatment severely inhibited OP mineralization in soil. For P transport/uptake and P deficiency responses, the BSi treatment showed a certain promoting effect compared with the other groups, while the effects of biochar and silicon-based conditioner applied alone showed little difference.

Based on the metagenomic analysis results of this study, the P-cycling functional genes (*gcd*, *ppx*, *pit*, *pstA*, *pstB*, *ugpB*, *ugpC*, *phoB*, and *phoR*) were consistently detected in both surface and subsurface layers across all treatments, with relative abundances >0.5‰ (Table 1). These genes constituted the core genetic basis of soil P metabolism under different amendment measures, consistent with the functional conservatism of soil P-cycling microbial communities reported in previous literature [35,36]. In the surface layer, the B treatment enhanced microbial-mediated P fixation and OP transformation processes, reducing the accumulation of soluble IP and thereby weakening its downward migration potential, thus promoting the retention of P in stable forms in the surface layer [37]. Although the BSi treatment promoted OP mineralization, it reduced Olsen-P content, possibly by accelerating the conversion of OP to moderately labile forms while simultaneously inhibiting P migration. In the subsurface soil, all treatments reduced the abundances of P-cycling-related genes, among which the B treatment exhibited the strongest inhibitory effect on IP-solubilization genes (e.g., *gcd* and *ppx*). The previous study demonstrated that biochar application significantly decreased the expression of the *gcd* gene encoding glucose dehydrogenase [37], a key enzyme in gluconic acid synthesis that is crucial for IP solubilization, leading to decreased microbial IP-solubilizing activity, reduced soluble P production in this layer, and thereby effectively retarding P leaching to deeper soils and groundwater.

Furthermore, the Si treatment significantly suppressed the expression of OP mineralization genes (e.g., *phoD*, *phoA*, and *phoX*), which can delay OP release and reduce the supply of leachable P [38]. Meanwhile, the BSi treatment showed a synergistic enhancement effect on genes related to P transport (*pstA/B*, *ugpB/C*) and P starvation response (*phoB/R*), enhancing microbial adaptive regulation to low-P conditions while maintaining phosphorus retention in the rhizosphere microdomain [37]. In summary, regulating the expression profiles of soil microbial P-cycling functional genes—particularly by inhibiting IP solubilization and optimizing OP mineralization pathways—is one of the key biological mechanisms affecting the potential for P leaching.

### 3.4. Environmental Factors—Microorganisms Involved in the P Cycle—The Interrelationship Between Environmental Factors and the P Cycle Process

**Figure 5:**
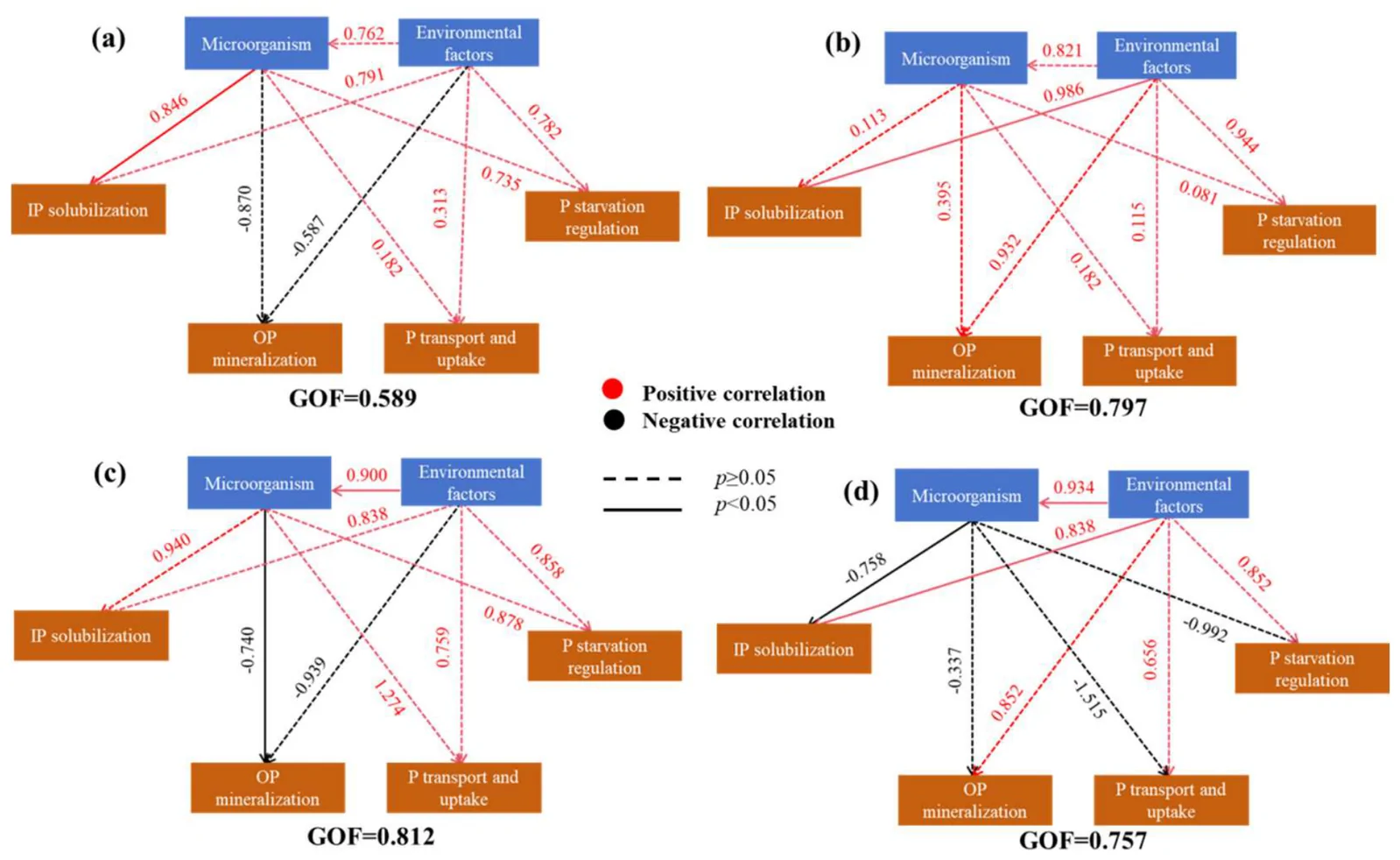
PLS-PM showing the effects of environmental factors and phylum-level microorganisms on P-cycling functional gene groups under different treatments (**a**) for CK, (**b**) for B, (**c**) for Si, and (**d**) for BSi. Note: sample size for each treatment-specific PLS-PM model: n = 6.

PLS-PM was used to analyze the interrelationships among environmental factors, soil P-cycling microorganisms, and P-cycling processes under different treatments in the purple-soil region (Figure 5a–d). The results showed that in the CK treatment, soil P-cycling microorganisms had a direct and significant positive effect on IP solubilization (*p* < 0.05), whereas environmental factors had no significant effect on IP solubilization (*p* ≥ 0.05). For OP mineralization, P transport and uptake, and P starvation regulation, neither soil P-cycling microorganisms nor environmental factors exerted significant effects (*p* ≥ 0.05 for all) (Figure 5a). In the B treatment, environmental factors exerted a direct and significant positive effect on IP solubilization (*p* < 0.05) (Figure 5b). In the Si treatment, environmental factors indirectly imposed a significant negative effect on OP mineralization by influencing microbial species abundance (*p* < 0.05) (Figure 5c). In the BSi treatment, environmental factors showed both a direct significant positive effect on IP solubilization and an indirect significant negative effect mediated through microbial species abundance (*p* < 0.05) (Figure 5d). These results suggest that optimizing fertilization management to improve soil environmental conditions may be more effective in inhibiting the transformation of IP into leachable P forms, thereby enhancing P retention capacity.

The PLS-PM analysis revealed the structured relationships among environmental factors, soil P-cycling microorganisms, and functional processes under different amendment measures. Under CK conditions, P-cycling microorganisms exhibited a significant direct positive effect on IP solubilization (*p* < 0.05), confirming the inherent dominance of microbially driven P mobilization in undisturbed purple soils [39]. In the B treatment, environmental factors showed a significant direct positive effect on IP solubilization (*p* < 0.05), indicating that biochar can promote the mobilization and dissolution of sparingly soluble IP by improving soil physicochemical properties. Concurrently, the porous structure and surface functional groups of biochar exert adsorption and fixation effects on dissolved P, forming a “dissolution–fixation” dynamic equilibrium with the IP dissolution process [34,38]. This equilibrium effectively retards downward P migration, thereby achieving a synergistic optimization of P bioavailability and environmental safety. Notably, in the Si treatment, environmental factors indirectly inhibited OP mineralization (*p* < 0.05) by significantly reducing the abundance of specific microbial taxa (e.g., *Bacillus* and *Pseudomonas*) [22], thus decreasing the production and downward migration of soluble P. In the BSi treatment, environmental factors exhibited both a direct positive effect and a microbial-mediated indirect negative effect on IP solubilization, suggesting an antagonistic regulation between the two pathways. On one hand, biochar can promote the release of phosphorus from Fe/Al oxides and sparingly soluble phosphates by increasing soil pH and through surface functional groups [38,40]; on the other hand, silicon-induced changes in microbial community structure (e.g., reducing the abundance of acid-producing P-solubilizing bacteria) may weaken this process [29]. Therefore, further studies are needed to identify the specific environmental factors and their effects on the abundance of key functional gene groups, so as to provide a theoretical basis for the precise regulation of the balance between soil phosphorus bioavailability and environmental risk.

**Figure 6:**
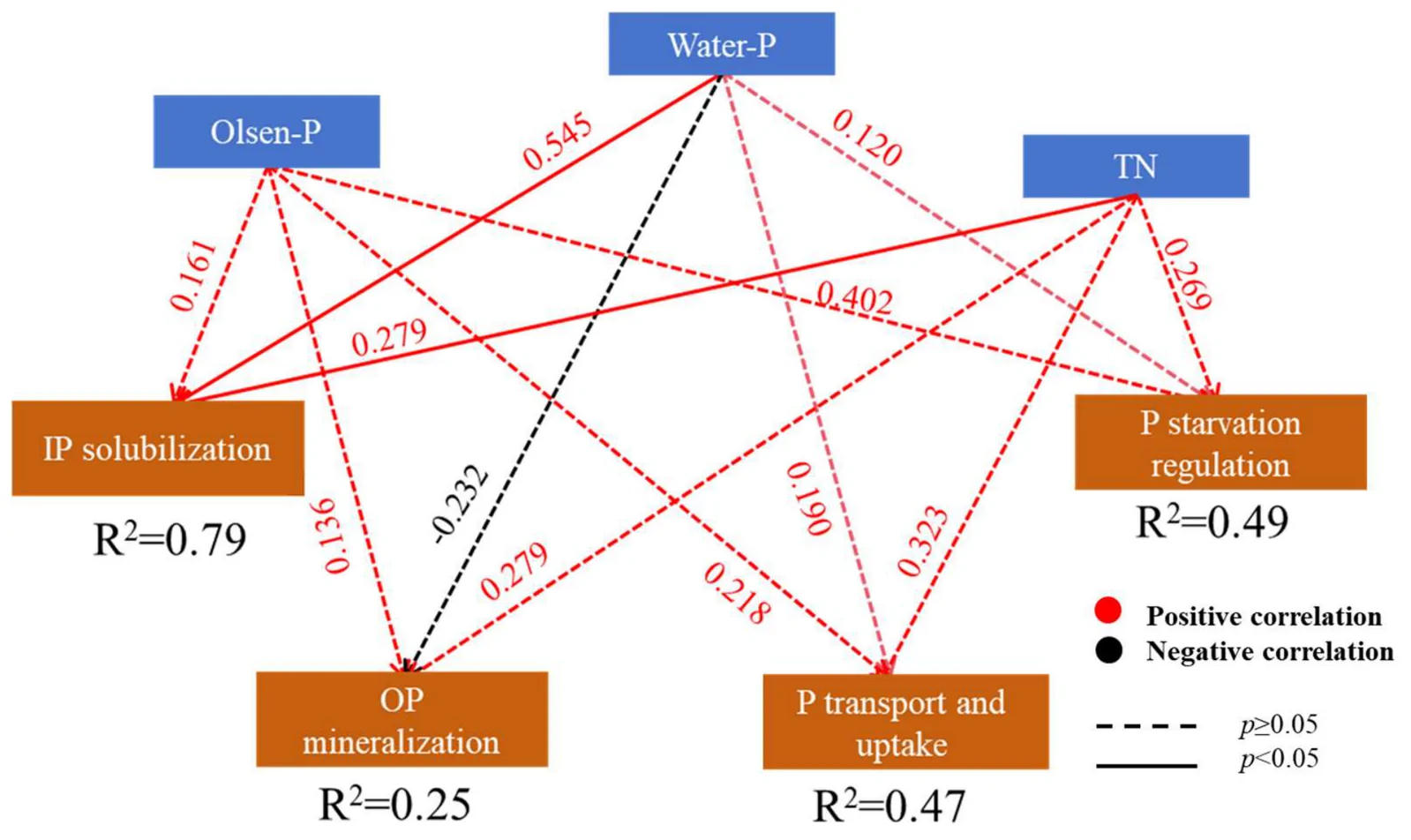
BSEM showing the effects of Olsen-P, Water-P, and TN on phosphorus-cycling functional gene groups. Values adjacent to arrows represent path coefficients. R^2^ indicates the proportion of variance explained for dependent variables. Note: sample size for the BSEM: n = 24.

Based on the results shown in Figure 5, a Bayesian structural equation model (BSEM) was constructed (Figure 6) to further explore the relationships between soil environmental factors (Olsen-P, Water-P, and TN) and the functional gene groups associated with the four P-cycling processes. Model diagnostic results indicated that the Rhat values for all parameters were <1.05, suggesting good model convergence; the effective sample sizes (ESS) for all parameters were >2000, ensuring the reliability of the estimates. The model results showed that the inorganic phosphorus solubilization process had the highest explanatory power (R^2^ = 0.79), with Water-P and TN contents exerting direct and significant positive effects on soil IP solubilization (Water-P: 95% CI = [0.19, 0.90]; TN: 95% CI = [0.02, 0.55]).

Based on the BSEM results shown in Figure 6, the statistical association patterns and underlying pathway mechanisms between soil Olsen-P, Water-P, and TN contents and the P-cycling functional gene groups were further elucidated. Water-P showed a significant positive correlation with the IP solubilization process, which is consistent with the underlying mechanisms of P leaching. As the main component of mobile phosphorus in soil solution, an increase in Water-P concentration generally reflects an elevated risk of P migration to deeper soil layers or runoff [41]. The synergistic positive effect of TN indicates that nitrogen supply improves microbial activity, thereby enhancing microbial processes related to P solubilization (e.g., organic acid secretion and proton release) [42]. Similar BSEM approaches have been successfully applied in soil P research to elucidate the complex pathways linking environmental factors to P-cycling processes. The positive effects of Water-P and TN on IP solubilization are consistent with previous findings that water-soluble P is a direct indicator of leaching risk, and that N supply enhances microbial P-solubilizing activity [38,41]. The high explanatory power (R^2^ = 0.79) is comparable to that reported in related soil P studies [42].

## 4. Conclusions

In conclusion, B applied alone proved most effective in simultaneously reducing P leaching and maintaining soil available P in dryland purple soils. Silicon alone was less effective, and the combined application of B and silicon was counterproductive, as silicon-induced P mobilization outweighed B-mediated retention under dryland aerobic conditions. Metagenomic analysis identified four functional microbial groups involved in soil P cycling—inorganic P solubilization, organic P mineralization, P transport and uptake, and P starvation regulation—with *gcd*, *ppx*, *pit*, *pstA/B*, *ugpB/C*, and *phoB/R* as the dominant functional genes across treatments. PLS-PM and BSEM analyses further revealed distinct regulatory pathways under different amendments: under CK, microorganisms directly drove IP solubilization; under B treatment, environmental factors were regulated to promote IP solubilization; under Si treatment, microbial species abundance was reduced to indirectly inhibit OP mineralization; and under BSi treatment, a bidirectional effect was observed—environmental factors promoting while microbial changes inhibiting IP solubilization. Additionally, Water-P and TN were identified as direct positive drivers of IP solubilization. Collectively, these findings establish that the key biological mechanisms for reducing P-leaching risk involve suppressing inorganic P solubilization in the subsurface layer, optimizing organic P mineralization in the surface layer, and enhancing P transport and starvation responses. This study provides a scientific basis for developing targeted P management strategies in dryland purple soils and highlights the value of integrating metagenomic approaches with path modeling to elucidate complex soil–microbe–environment interactions.

## Figures and Tables

**Table 1:** Names of functional genes with phosphorus cycle.

Functional Groups	Genes
IP solubilization	*gcd*, *ppx*
OP mineralization	*phoD*, *phoA*, *phoX*, *ugpQ*, *phnJ*, *phnX*
P transport and uptake	*Pit*, *pstA*, *pstB*, *ugpB*, *ugpC*
P starvation regulation	*phoB*, *phoR*

## Data Availability

The original contributions presented in this study are included in the article; further inquiries can be directed to the corresponding author.

## Supplementary Materials

The following supporting information can be downloaded at: https://www.mdpi.com/article/10.3390/biology15181658/s1, Table S1: Properties of the soil used in the experiment. Table S2: Basic properties of the biochar and silicon-based conditioner used in the experiment. Table S3: Details of experimental design. Figure S1: Schematic diagram of the indoor P leaching experiment.
