# Supplementary material for: Study on the Risk of Phosphorus Leaching in Dryland from Typical Purple-Soil Regions and Its Control Mechanisms
Source: Biology (Basel). 2026 Sep 19;15(18):1658. doi: 10.3390/biology15181658 (PMC13604246; doi:10.3390/biology15181658)

Supplementary Materials

Study on the Risk of Phosphorus Leaching in Dryland from Typical Purple-Soil Regions and Its Control Mechanisms

Xiaosong Yang [1], Jingwen Yu [2], Yanfen Wang [1,3], Yiming Zhao [1,3], Kun Wang [1], Xiaofeng Lu [3,*] and Lan Zhang [1,*]

1 State Key Laboratory of Environmental Criteria and Risk Assessment, National Engineering Laboratory of Lake Water Pollution Control and Ecological Restoration Technology, Chinese Research Academy of Environmental Sciences, Beijing 100012, China; yangxiaosong15@mails.ucas.ac.cn (X.Y.); 19863962513@163.com (Y.W.); 15776814368@163.com (Y.Z.); wangkun@craes.org.cn (K.W.)

2 Yangtze Ecology and Environment Co., Ltd., Wuhan 430014, China; wei_jingwen@ctg.com.cn

3 College of Water Conservancy, Shenyang Agricultural University, Shenyang 110866, China

* Correspondence: 13654906041@163.com (X.L.); zhanglan3524@163.com (L.Z.)

Table S1 Properties of the soil used in the experiment

Depth (cm)	pH	TN (g/kg)	DOC (mg/kg)	TP (g/kg)	Olsen-P (mg/kg)	Water-P (mg/kg)	Volume weight (g/cm^3)
0-20 cm	5.4	1.07	78.5	0.47	5.000	2.71	1.22
20-40 cm	5.6	0.88	122.3	0.35	7.18	2.50	1.24

Table S2 Basic properties of the biochar and silicon-based conditioner used in the experiment

**Conditioner**	TN (g/kg)	DOC (mg/kg)	TP (g/kg)	Olsen-P (mg/kg)	Water-P (mg/kg)
C	15.68	1250.00	0.83	61.24	38.78
Si	2.10	0.80	0.51	45.44	51.49

Table S3 Details of experimental design

	Si	C	Nitrogen application	Phosphorus application
CK	0	0	178.5 kg N/hm^2	90 kg P_2O_5/hm^2
Si	30 t/hm^2	0	178.5 kg N/hm^2	90 kg P_2O_5/hm^2
B	0	30 t/hm^2	178.5 kg N/hm^2	90 kg P_2O_5/hm^2
BSi	30 t/hm^2	30 t/hm^2	178.5 kg N/hm^2	90 kg P_2O_5/hm^2

Figure S1 Schematic diagram of the indoor P leaching experiment

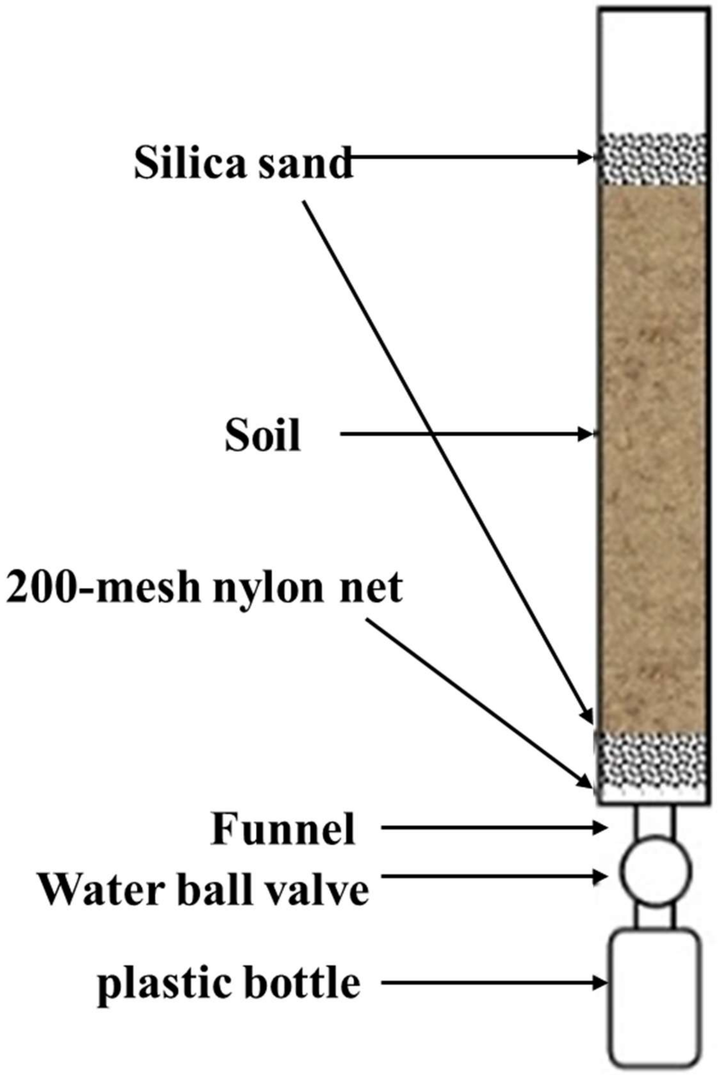

Supplement: Supplementary file 1 [file biology-15-01658-s001.zip › biology-4522541-supplementary.pdf]
